# Supplementary figures and images for: Foot-and-Mouth Disease Virus Serotype O Exhibits Phenomenal Genetic Lineage Diversity in India during 2018–2022
Source: Viruses. 2023 Jul 10;15(7):1529. doi: 10.3390/v15071529 (PMC10384687; doi:10.3390/v15071529)

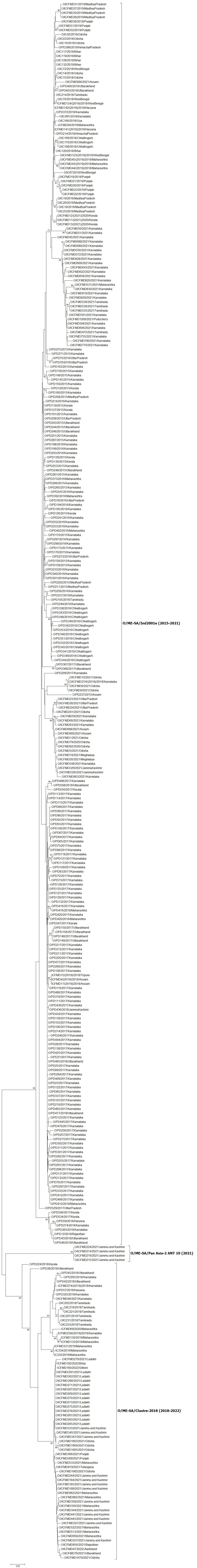

Supplement: Supplementary file 1 [file viruses-15-01529-s001.zip › Fig S1.png]
